# Optimizing grid-connected PV systems with novel super-twisting sliding mode controllers for real-time power management

**DOI:** 10.1038/s41598-024-55380-3

**Published:** 2024-02-26

**Authors:** Bhabasis Mohapatra, Binod Kumar Sahu, Swagat Pati, Mohit Bajaj, Vojtech Blazek, Lukas Prokop, Stanislav Misak

**Affiliations:** 1 Department of Electrical Engineering, ITER, Siksha ‘O’ Anusandhan (Deemed to be University), Bhubaneswar, Odisha India; 2 Department of Electrical Engineering, Graphic Era (Deemed to be University), Dehradun, 248002 India; 3 Hourani Center for Applied Scientific Research, Al-Ahliyya Amman University, Amman, Jordan; 4 Graphic Era Hill University, Dehradun, 248002 India; 5 Applied Science Research Center, Applied Science Private University, Amman, 11937 Jordan; 6 ENET Centre, VSB—Technical University of Ostrava, 708 00 Ostrava, Czech Republic

**Keywords:** Improved arithmetic optimization algorithm (IAOA), Particle swarm optimization (PSO), Super twisting sliding mode controller (ST-SMC), Proportional-integral (PI) controller, Conventional arithmetic optimization algorithm (CAOA), Grid connected photovoltaic system (GCPV), Photovoltaic (PV), Energy science and technology, Engineering

## Abstract

Over the past years, the use of renewable energy sources (RESs) has grown significantly as a means of providing clean energy to counteract the devastating effects of climate change. Reducing energy costs and pollution have been the primary causes of the rise in solar photovoltaic (PV) system integrations with the grid in recent years. A load that is locally connected to a GCPV requires both active and reactive power control. In order to control both active and reactive power, MAs and advanced controllers are essential. Researchers have used one of the recently developed MAs, known as the CAOA, which is based on mathematical arithmetic operators to tackle a few real-world optimization problems. Some disadvantages of CAOA include its natural tendency to converge to a local optimum and its limited capacity for exploration. By merging the PSO and CAOA methodologies, this article suggests the IAOA. To show how applicable IAOA is, its performance has been evaluated using four benchmark functions. The implementation of an IAOA-based ST-SMC for active and reactive power control is addressed in this article, which offers an innovative approach of research. In comparison to PSO-based ST-SMC and CAOA-based ST-SMC, the proposed IAOA-based ST-SMC appears to be superior, with settling time for active and reactive power control at a minimum of 0.01012 s and 0.5075 s. A real-time OPAL-RT 4510 simulator is used to validate the performance results of a 40 kW GCPV system after it has been investigated in the MATLAB environment.

## Introduction

The on-going shift of the global energy landscape highlights the importance of sources of clean energy in supporting sustainability and minimising the impact on the environment^1,2^. Among them, PV systems have the greatest promise for producing clean, plentiful energy. The photovoltaic technology is employed for the generation of electricity, which is supplied to the utility grid for consumer utilization^3,4^. The eco-friendliness and depletion of fossil fuels have increased the demand for PV systems. The intermittent nature of the sunlight has made PV technology a less reliable source of energy^5^. Electricity consumers also face a high installation cost and low power conversion efficiency in PV systems^6^. As a result, the development of power electronics technology increases system efficiency and offers possibilities for using the PV source in different applications^7,8^. Integrating PV systems into the electrical grid demands sophisticated control techniques in order to maximise their performance^9,10^.

A thorough assessment of available literature illustrates the changing landscape of control techniques used in grid-connected PV systems. Conventional control approaches, which frequently depend on PI controllers, are limited in their ability to adapt to the dynamic and nonlinear features of PV systems^11,12^. The limits of PI controllers, particularly their sensitivity to significant early undershoots, have caused a rethinking of control paradigms. As a result, sophisticated control techniques to improve the accuracy and robustness of power regulation are clearly needed^13,14^. The rapid developments in various application fields have made optimization problems more complex. Traditional optimization methods, for instance Incremental Conductance (IC) and Perturb and Observe (P&O), are recommended in Zhang et al.^15^ and Subudhi and Pradhan^16^. Conventional optimization techniques require more cost and time. The MAs have proved to be more reliable than the conventional methods in the recent era^17,18^. MAs like Grey Wolf Optimization (GWO)^19,20^, Ant Colony Optimization (ACO) and Artificial Bee Colony (ABC)^21,22^ are examples in the field. With quick development in the design of MAs, researchers have suggested improved algorithms such as WQSMA^23^, JSWOA^24^, DSCA^25^, and EHHO^26^. The upgraded MAs address optimization issues more effectively than conventional MAs.

In order to estimate transmission line parameters, Shaikh et al.^27^ proposed a hybrid moth-flame optimization (MFO) and PSO approach. This method was validated mathematically on a variety of benchmark functions and was based on many scenarios. This algorithm logically incorporates the ideas of MFO and PSO to get beyond their drawbacks and enhance their capacity for global search. The results indicated that the proposed hybrid algorithm outperforms the original MFO and conventional PSO in terms of convergence speed and solution quality. Shaikh et al.^28^ developed an enhanced moth flame optimization technique for parameters estimation of AC transmission lines. The outcomes of the suggested approach are contrasted with those of alternative approaches. The comparison demonstrates how quickly and precisely the proposed approach converges to the best-obtained value. The enhanced moth flame optimization outperforms the previous methods in terms of practicality and solution quality, demonstrating its efficacy. A modified whale optimization algorithm (MWOA) based on levy flying was presented by Shaikh et al.^29^ to compute the overhead AC transmission line parameters from four perspectives: dimension selection, exploration controls, modified encircling prey, and candidate solution determining. For single-phase and three-phase applications with varying numbers of bundle conductors, the proposed work offered improved whale optimization techniques to compute capacitance and inductance per unit length. The MWOA technique yields more accurate and reliable global or near-global optimal control variable settings, as demonstrated by the results. To determine the parameters of the transmission line, Shaikh et al.^30^ recommended employing the flux linkage technique. Finding the transmission-line parameters and analysing the impact of various bundle conductor configurations—such as two, three, and four bundle conductors—were the goals of this research work. The presented simulation results demonstrate the effectiveness of the suggested strategies under a range of bundle conductor configurations. In order to determine the overhead transmission line parameter, Shaikh et al.^31^ devised GWO, a novel optimisation technique. When compared to earlier algorithms, the outcomes of the GWO algorithms are superior and optimized. The suggested algorithm outperforms the others in terms of accuracy, robustness, and convergence speed and is computationally efficient, in accordance with the results. Shaikh et al.^32^ put forwarded the application of a WOA to determine the overhead AC transmission line characteristics. Single-phase and three-phase AC transmission lines are used with the method suggested. Using suggested WOA methods for various bundle conductor arrangements, this research work has calculated capacitance and inductance per unit length for three-phase. Nevertheless, an outline of the load modelling study for voltage stability is also included in this research work.

Abualigah et al. familiarized an innovative meta-heuristic algorithm acknowledged as CAOA, which leverages elementary mathematical operations including Addition (A), Multiplication (M), Subtraction (S), and Division (D)^33^. Abualigah et al. suggested an approach inspired from the combination of CAOA and the differential evolution algorithm to increase capability of searching^34^. Manoharan et al. devised a multi-objective arithmetic optimization Algorithm (MOAOA) technique to tackle limited multi-objective optimization challenges^35^. Khatir et al.^36^ offered a CAOA-based improved artificial neural network for functionally graded material (FGM) plate constructions. The CAOA technique's fundamental shortcoming is an improper balance between exploration and exploitation throughout the search phase. In many cases, the CAOA technique gets trapped in local optima. Therefore, this article proposes an IAOA algorithm by combining Particle Swarm Optimization with CAOA for the GCPV system. As a result, the IAOA technique outperforms other techniques in terms of settling time and % undershoot for active and reactive power regulation with fewer iterations. Some standard benchmark functions are taken into consideration for testing the proposed IAOA technique.

Various control techniques such as PI controller, SMC and ST-SMC are employed for effective management of active and reactive power in a three-phase GCPV system. The PI controller is used by most of the researchers due to moderate cost, simplicity and applicability, but the major drawback of this controller is less sensitivity to parameter changes and outside disturbances. In order to bring back the state trajectory of a system towards the sliding surface, SMC is employed. The major disadvantage of SMC is chattering^37^. High-order sliding mode controllers are used as substitutes for the SMC to reduce the chattering^38^.

A novel super-twisting adaptive sliding mode law was proposed by Shtessel et al.^39^ to control electro pneumatic actuators. Preserving the accuracy of the control gain magnitude is a crucial aspect of the adaptive method. A multivariable super-twisting sliding mode structure that expands on the well-known single-input instance was presented by Nagesh et al.^40^. Yang et al.^41^ presented an adaptive super-twisting sliding mode controller (SSMC) with output feedback for hydraulic systems with unmodeled disturbances using an extended state observer (ESO). In order to address uncertainty, Gurumurthy et al.^42^ developed an adaptive super twisting sliding mode controller (ASTSMC). A continuous-time robust reference model was put forward by Hollweg et al.^43^, and its stability is studied using STSMC and Lyapunov stability theory. For patients with type 1 diabetes, Ahmad et al.^44^ introduced a closed-loop control strategy based on SMC control. Recently, ST-SMC^45^ has gained research interest in grid-connected PV systems due to their flexibility and effectiveness in providing appropriate control action. In the case of ST-SMC, additional information about the system is not mandatory. The ST-SMC has the capability to reduce the chattering effect in the presence of finite-time convergence to the sliding surface, system robust stabilization and uncertainties, which makes it a benchmark for the second-order SMC. The application of an optimally tuned ST-SMC technique has been reported to have a better dynamic response than conventional PI and SMC controllers^46^.

The present work tackles crucial issues in the regulation of grid-connected Photovoltaic systems, where conventional PI controllers fall short because of significant early undershoots. Furthermore, chattering, an unwanted fast oscillation in control signals, is a problem with the traditional Sliding Mode Control method, despite it being robust. Surpassing PI controller constraints, reducing SMC chattering, and optimizing control parameters are the main objectives. Modern arithmetic optimization algorithms are incorporated to fine-tune control parameters in order to do this, including PSO, CAOA, and Improved Arithmetic Optimization Algorithm (IAOA). This study leads to the development of an Optimally Tuned Super-Twisting Sliding Mode Controller, which employs sophisticated optimisation algorithms to provide improved management of active and reactive power in grid-connected PV systems. This comprehensive strategy helps to the advancement of renewable energy control systems by providing innovative solutions to the complexity inherent in PV system dynamics.

They key contributions of the paper are as follows:

i. Optimization-Driven Control Parameters: Introduces a novel method for optimizing the parameters of the ST-SMC by using advanced arithmetic optimization algorithms, such as PSO, CAOA, and IAOA. The accuracy and responsiveness of the control system are greatly improved by this optimization.
ii. Overcoming PI Controller Limitations: Addresses the drawbacks of conventional PI controllers, particularly the issue of significant early undershoots in the control of reactive and active power. The suggested approach offers better performance as a viable substitute for traditional PI controllers.
iii. Chattering Mitigation: Identifies and addresses the chattering problem via SMC approaches. The study helps to lessen chattering effects, providing smoother and more reliable control signals by utilizing the Super-Twisting algorithm and higher-order sliding mode approaches.
iv. Real-Time Validation: Real-time testing on an OPAL-RT 4510 platform are used to validate the proposed optimally tuned ST-SMC. This real-world validation illustrates the proposed control strategy's practical applicability and efficacy.
v. Comparative Performance Analysis: Performs a detailed comparison of the proposed ST-SMC controller tuned by several optimization methods (PSO, CAOA, IAOA) under diverse scenarios. This study gives useful insights into the strengths and shortcomings of each algorithm, assisting both researchers and developers in identifying the best optimization strategy for comparable applications.

The paper is organised in sections, beginning with an explanation of the system framework in Section "System Framework" and ending with a detailed explanation of the system design in Section "System Design". Section "Control Structure" presents the control structure's complexities, elucidating its components and functions. Section "Optimization Techniques and Benchmark Function’s" focuses on the optimisation technique and the investigation of various benchmark functions. The following section, Section "Design of Controllers", meticulously outlines the controller's design aspects. Section "Results and Discussion" represents the results and discussion section of the experimental findings. Finally, in Section "Conclusion and Future Research Directions", the conclusive insights and outcomes are encapsulated, providing a comprehensive conclusion to the article.

## System framework

**Figure 1:**
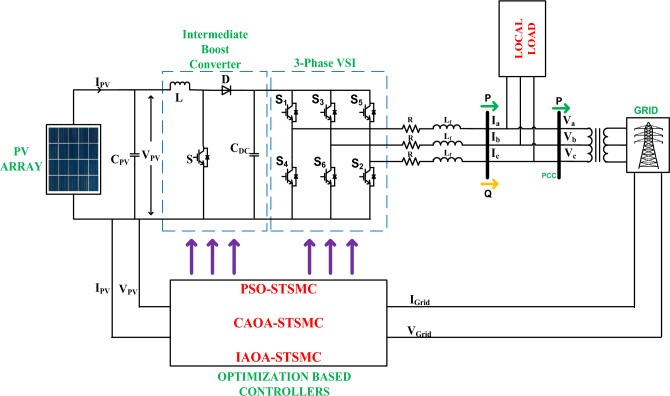
A proposed three-phase GCPV system structure.

The structure of a three-phase Grid-Connected Photovoltaic system is depicted in Fig. 1. The system framework basically comprises a PV array, intermediate boost converter (IBC), optimally tuned controllers and local load. The proposed control structure regulates both active and reactive power. The suggested control configuration comprises an orthogonal current component, that is additional subdivided into two components: the in-phase component and the quadrature component. The active power is managed by the in-phase component, while the reactive power is managed by the quadrature component. Table 1 tabulates the simulation parameters for the study. The converter has a 50 kVA output voltage with 1000 V DC-link voltage. The parameters P_ref_ (active power reference) and Q_ref_ (reactive power reference) undergo variations within the range of 12 kW to 20 kW and '0' kVAR to 14 kVAR, respectively.

**Table 1:** Specifications for simulation.

Parameter	Numerical-value
PV system’s power rating	40 kW
No. of parallel panels	20
No. of series panels	10
Short-circuit current of each panel	8.21 A
Open-circuit voltage of each panel	32.9 V
Parallel-resistance	415.4 Ω
Series-resistance	0.221 Ω
Total panels number	200
Voltage at DC-link	1000 V
Locally connected load	10 kW, 2 kVAR
Operating frequency range	10 kHz
Inverter KVA	50
Irradiance	1000 W/m^2^

## System design

The proposed system has a power rating of 40 kW, a 50 kVA inverter, and a local load of 10 kW. The following equations illustrate the calculations for the line current, dc-link voltage, current ripple and inductance specifications of the proposed GCPV system.

Equation (1) shows the calculation of the line current (I_S_):

1 $${{\text{I}}}_{{\text{S}}}=\frac{{\text{S}}}{{\sqrt{3}{\text{V}}}_{{\text{L}}}}=69.56\mathrm{ A}$$

The rated voltage at no load is 415 V_L-L_. To achieve satisfactory Pulse Width Modulation (PWM) control, the DC bus voltage must exceed the peak of the line voltage. The direct current link voltage is as follows:

2 $$ {\text{V}}_{{{\text{dc}}}} = \frac{{2\sqrt 2 \left( {\frac{{\text{V}}}{\sqrt 3 }} \right)}}{{{\text{m}}_{{\text{a}}} }} = { 847}\;{\text{ V }} \approx {1}000 \, \;{\text{V }}\left( {{\text{selected}}} \right) $$

where modulation index (m_a_) is taken as 0.8.

The direct current link voltage is assumed to be 1000 V, a slightly higher rounded number. 5% of I_S_ is considered as a Current ripple (i_cr_) through the inductor.

Equation (3) shows the inductance calculation

3 $$ {\text{L}} = \frac{{\left( {\frac{\sqrt 3 }{2}} \right){*}0.8{\text{*V}}_{{{\text{dc}}}} }}{{6{\text{*a*f}}_{{\text{s}}} {\text{*i}}_{{{\text{cr}}}} }} = \, 0.{78 }\;{\text{mH }} \approx { 1 }\;{\text{mH}} $$

where f_s_ is switching frequency chosen to be 10 kHz. During transients, a current rating of 150% (a = 1.5) of steady-state current.

The calculation of voltage drop across the inductor is as follows

4 $$ = { 2}\pi *{\text{ f}}*{\text{L}}*{\text{I}}_{{\text{S}}} = { 21}.{\text{85 V}},{\text{ which is 5}}.{2}\% {\text{ of V}}_{{\text{L}}} . $$

## Control structure

**Figure 2:**
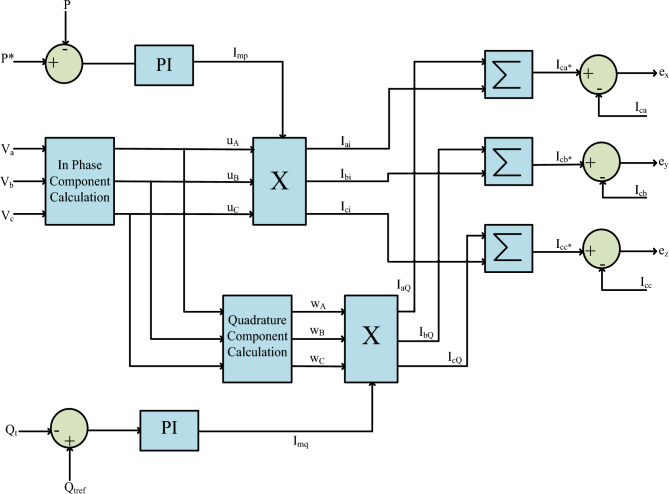
Active and reactive power control structures.

The regulating structure shown in Fig. 2 manages the active and reactive power. Equation (5) shows the peak voltage as^47^:

5 $${{\text{V}}}_{{\text{T}}}=\sqrt{\frac{2}{3}\left({{\text{V}}}_{{\text{A}}}^{2}+{{\text{V}}}_{{\text{B}}}^{2}+{{\text{V}}}_{{\text{C}}}^{2}\right)}$$

The in-phase components are given in Eq. (6).

6 $${{\text{u}}}_{{\text{A}}}=\frac{{{\text{V}}}_{{\text{A}}}}{{{\text{V}}}_{{\text{T}}}}, {{\text{u}}}_{{\text{B}}}=\frac{{{\text{V}}}_{{\text{B}}}}{{{\text{V}}}_{{\text{T}}}}, {{\text{u}}}_{{\text{C}}}=\frac{{{\text{V}}}_{{\text{C}}}}{{{\text{V}}}_{{\text{T}}}}$$

The quadrature components (w_A_, w_B_, w_C_) are determined in the following way:

7 $$\left[\begin{array}{c}{{\text{w}}}_{{\text{A}}}\\ {{\text{w}}}_{{\text{B}}}\\ {{\text{w}}}_{{\text{C}}}\end{array}\right]=\left[\begin{array}{ccc}0& -\frac{1}{\sqrt{3}}& \frac{1}{\sqrt{3}}\\ \frac{\sqrt{3}}{2}& \frac{1}{2\sqrt{3}}& -\frac{1}{2\sqrt{3}}\\ -\frac{\sqrt{3}}{2}& \frac{1}{2\sqrt{3}}& -\frac{1}{2\sqrt{3}}\end{array}\right]\left[\begin{array}{c}{{\text{u}}}_{{\text{A}}}\\ {{\text{u}}}_{{\text{B}}}\\ {{\text{u}}}_{{\text{C}}}\end{array}\right]$$

The STSMC controller obtains and handles active and reactive power measurement errors.

8 $${{\text{I}}}_{{\text{up}}}\left({\text{n}}\right)={{\text{I}}}_{{\text{up}}}\left({\text{n}}-1\right)+{{\text{K}}}_{{\text{iu}}}{{\text{e}}}_{{\text{rrf}}}\left({\text{n}}\right)+{{\text{K}}}_{{\text{pu}}}\left({{\text{e}}}_{{\text{rrf}}}\left({\text{n}}\right)-{{\text{e}}}_{{\text{rrf}}}\left({\text{n}}-1\right)\right)$$

9 $${{\text{I}}}_{{\text{wp}}}\left({\text{n}}\right)={{\text{I}}}_{{\text{wp}}}\left({\text{n}}-1\right)+{{\text{K}}}_{{\text{iw}}}{{\text{e}}}_{{\text{rrv}}}\left({\text{n}}\right)+{{\text{K}}}_{{\text{pw}}}\left({{\text{e}}}_{{\text{rrv}}}\left({\text{n}}\right)-{{\text{e}}}_{{\text{rrv}}}\left({\text{n}}-1\right)\right)$$

where,

10 $${{\text{e}}}_{{\text{rrf}}}={{\text{P}}}^{*}-{\text{P}}$$

11 $${{\text{e}}}_{{\text{rrv}}}={{\text{Q}}}_{{\text{tref}}}-{{\text{Q}}}_{{\text{t}}}$$

where 'P*' is the grid's reference active power, 'Q_tref_' is the grid's reference reactive power, 'Q_t_' is the reactive power, and 'P' is the grid's active power.

In-phase and quadrature current components are I_up_ and I_wp,_ respectively. After multiplying with their respective amplitudes, the quadrature current and in-phase components are as follows:

12 $$ \begin{aligned} {\text{I}}_{{{\text{wa}}}} & = {\text{I}}_{{{\text{wp}}}} {\text{*w}}_{{\text{a}}} \\ {\text{I}}_{{{\text{wb}}}} & = {\text{I}}_{{{\text{wp}}}} {\text{*w}}_{{\text{b}}} { } \\ {\text{I}}_{{{\text{wc}}}} & = {\text{I}}_{{{\text{wp}}}} {\text{*w}}_{{\text{c}}} \\ \end{aligned} $$

13 $$ \begin{aligned} {\text{I}}_{{{\text{ua}}}} & {\text{ = I}}_{{{\text{up}}}} {\text{*u}}_{{\text{a}}} \\ {\text{I}}_{{{\text{ub}}}} & {\text{ = I}}_{{{\text{up}}}} {\text{*u}}_{{\text{b}}} \\ {\text{I}}_{{{\text{uc}}}} & {\text{ = I}}_{{{\text{up}}}} {\text{*u}}_{{\text{c}}} \\ \end{aligned} $$

In Eq. (14), the three-phase reference currents are determined.

14 $$ \begin{aligned} {\text{i}}_{{\text{a}}}^{*} & = {\text{i}}_{{{\text{ua}}}} + {\text{i}}_{{{\text{wa}}}} \\ {\text{i}}_{{\text{b}}}^{*} & = {\text{i}}_{{{\text{ub}}}} + {\text{i}}_{{{\text{wb}}}} \\ {\text{i}}_{{\text{c}}}^{*} & = {\text{i}}_{{{\text{uc}}}} + {\text{i}}_{{{\text{wc}}}} \\ \end{aligned} $$

The actual currents are subtracted from their respective reference current values to get the control signals ‘e_x_’, ‘e_y_’, ‘e_z_’.

15 $$ \begin{aligned} {\text{e}}_{{\text{x}}} & = {\text{i}}_{{\text{a}}}^{*} - {\text{i}}_{{\text{a}}} \\ {\text{e}}_{{\text{y}}} & = {\text{i}}_{{\text{b}}}^{*} - {\text{i}}_{{\text{b}}} \\ {\text{e}}_{{\text{z}}} & = {\text{i}}_{{\text{c}}}^{*} - {\text{i}}_{{\text{c}}} \\ \end{aligned} $$

## Optimization techniques and benchmark function’s

### Particle swarm optimization (PSO)

Kennedy and Eberhard introduced particle swarm optimization in 1995^21^, which was influenced by natural occurrences. The following are the PSO stages:

i. Initialization: A starting population and velocity of size [NP × D] are generated within the specified search range. At this point, “NP” denotes the population number, and "D" represents the problem dimension^48^.

ii. Updating the velocity: The velocity is updated according to Eq. (16)

16 $${{\text{v}}}_{{\text{new}}}={\text{w}}\times {{\text{v}}}_{{\text{old}}}+{{\text{C}}}_{1}\times {{\text{rand}}}_{1}\times \left({{\text{p}}}_{{\text{best}}}-{\text{x}}\right)+{{\text{C}}}_{2}\times {{\text{rand}}}_{2}\times \left({{\text{g}}}_{{\text{best}}}-{\text{x}}\right)$$

where “C_1_” and “C_2_” are acceleration constants that are commonly taken to be 2 and.05, “g_best_” is the global best, “p_best_” is the local best, “rand_1_” and “rand_2_” are random values between [0, 1], and “w” is the inertia weight, which decreases linearly from 0.9 to 0.4.

iii. Updating the position: The initial population experiences an update by uniting it with the newly generated velocity^49,50^.

17 $${{\text{x}}}_{{\text{new}}}={{\text{x}}}_{{\text{old}}}+{{\text{v}}}_{{\text{new}}}$$

### Forensic-based investigation (FBI) algorithm

**Figure 3:**
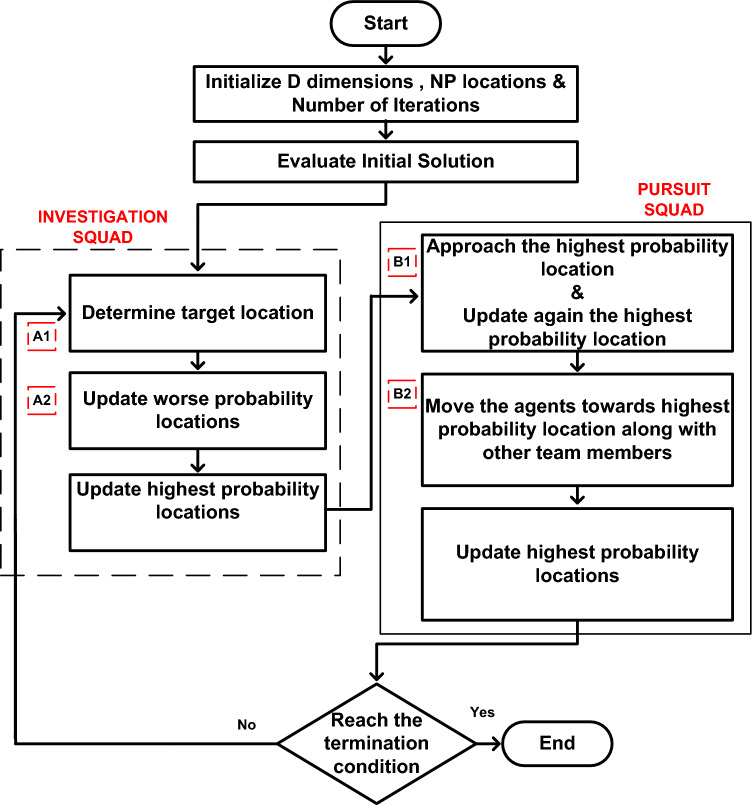
General Principle of FBI algorithm.

The FBI was developed by Chou and Nguyen^51^ and is based on three basic concepts: location, suspected investigation, and stalking. There are five steps in an extensive forensic investigation process: opening the case, analysing findings, and looking at directions, actions, and trials. The police squad gathers information on the crime initially, and this information assists them in starting the investigation^52,53^. The investigative team looks at the scene of the crime, prospective suspects, the victim, and the evidence of the crime. By examining the data and contrasting it with the perceptions gathered throughout the investigation, the team determines who are the most likely the culprits. The flowchart of the FBI is depicted in Fig. 3.

Suspected locations are searched by the investigating officers to trace out the possible locations of hiding. A search team is organized to identify the location of the suspect with highest possibility. In order to arrest the criminal, the police team move towards the traced location. The information regarding the traced location is informed to the police headquarters. The pursuit team and the investigation team coordinate each other about the findings, investigation and next approach about the case^54,55^. The pursuit team direct reports to the investigating team about the matter so that the suspect can be detected easily. The five stages are as follows.

(i) Case Opening: The crime scene is investigated by the police officers and collects the information. The preliminary idea is gained by the members of the team who initially investigated the scene by considering various standard operating procedures. The team questions witnesses and locates the witnesses.
(ii) Findings from Interpretation: The overall information is gained by the members of the team through team meetings. The possible suspects of the concerned case are evaluated by assessing the information in the team briefings.
(iii) Inquiry direction: Based on the analysis of findings from various theories are developed i.e. crime motives, inquiry lines and scenarios by the members of the team. The new theories are again evaluated through investigations and are compared with the old theories for better interpretation of the case.
(iv) Course of action: The members of the team set the priorities and inquiry lines for taking actions. New analysis comes out from the actions taken. The actions and inquiry directions are again refined through new outcomes of the case.
(v) Trial: The final process where fair and dominating outcome of the case comes out. A severe suspect is identified and the prosecution takes place. Depending upon the complexity, severity and difficulty of the case the investigating police officers are decided. Depending upon the characteristics of the case, the total numbers of investigating officers may be tens, hundreds or thousands.

Steps involved in FBI Algorithm:

**Step A**_**1**_**:** The 'investigation findings' are represented in this step.

18 $${{\text{X}}}_{{{\text{A}}}_{{\text{ij}}}}^{\mathrm{^{\prime}}}={{\text{X}}}_{{{\text{A}}}_{{\text{ij}}}}+ \frac{{\text{rand}}* \left({\sum }_{1}^{{{\text{a}}}_{1}}{{\text{X}}}_{{{\text{A}}}_{{\text{aj}}}}\right)}{{{\text{a}}}_{1}}$$

19 $${{\text{X}}}_{{{\text{A}}}_{{\text{ij}}}}^{\mathrm{^{\prime}}}= {{\text{X}}}_{{{\text{A}}}_{{\text{ij}}}}+\mathrm{ rand}* \frac{{{\text{X}}}_{{{\text{A}}}_{{\text{ij}}}}- \left({{\text{X}}}_{{{\text{A}}}_{{\text{kj}}}}+ {{\text{X}}}_{{{\text{A}}}_{{\text{hj}}}}\right)}{2}$$

**Step A**_**2**_**:** The 'inquiry directions' are presented in this step.

20 $$\mathrm{Prob }\left({{\text{X}}}_{{{\text{A}}}_{{\text{ij}}}}\right)= \left({{\text{p}}}_{{{\text{A}}}_{{\text{i}}}}- {{\text{p}}}_{{\text{min}}}\right)/ \left({{\text{p}}}_{{\text{max}}}- {{\text{p}}}_{{\text{min}}}\right)$$

21 $${{\text{X}}}_{{{\text{A}}}_{{\text{ij}}}}^{\mathrm{^{\prime}}}={{\text{X}}}_{{\text{min}}}+ {\sum }_{1}^{{{\text{a}}}_{2}}{\mathrm{\alpha }*{\text{X}}}_{{{\text{A}}}_{{\text{bj}}}}$$

22 $${{\text{X}}}_{{{\text{A}}}_{{\text{ij}}}}^{\mathrm{^{\prime}}}={{\text{X}}}_{{\text{min}}}+ {{\text{X}}}_{{{\text{A}}}_{{\text{dj}}}}+\mathrm{ rand}* \left({{\text{X}}}_{{{\text{A}}}_{{\text{kj}}}}- {{\text{X}}}_{{{\text{A}}}_{{\text{hj}}}}\right)$$

**Step B**_**1**_: The 'actions' taken in the investigation are represented in this step.

23 $${{\text{X}}}_{{{\text{B}}}_{{\text{ij}}}}^{\mathrm{^{\prime}}}= {{\text{rand}}1*{\text{X}}}_{{{\text{B}}}_{{\text{ij}}}}+\mathrm{ rand}2*\left({{\text{X}}}_{{\text{min}}}- {{\text{X}}}_{{{\text{B}}}_{{\text{ij}}}}\right)$$

**Step B**_**2**_: The 'process of actions' is illustrated in this step.

24 $${{\text{X}}}_{{{\text{B}}}_{{\text{ij}}}}^{\mathrm{^{\prime}}}= {{\text{X}}}_{{{\text{B}}}_{{\text{rj}}}}+\mathrm{ rand}3*\left({{\text{X}}}_{{{\text{B}}}_{{\text{rj}}}}- {{\text{X}}}_{{{\text{B}}}_{{\text{ij}}}}\right)+\mathrm{ rand}4*\left({{\text{X}}}_{{\text{min}}}- {{\text{X}}}_{{{\text{B}}}_{{\text{rj}}}}\right)$$

25 $${{\text{X}}}_{{{\text{B}}}_{{\text{ij}}}}^{\mathrm{^{\prime}}}= {{\text{X}}}_{{{\text{B}}}_{{\text{ij}}}}+\mathrm{ rand}3*\left({{\text{X}}}_{{{\text{B}}}_{{\text{ij}}}}- {{\text{X}}}_{{{\text{B}}}_{{\text{rj}}}}\right)+\mathrm{ rand}4*\left({{\text{X}}}_{{\text{min}}}- {{\text{X}}}_{{{\text{B}}}_{{\text{ij}}}}\right)$$

where,

**Table:** 

${{\text{X}}}_{{{\text{A}}}_{{\text{ij}}}}$= Suspected location	rand is a random number in the range [− 1, 1]
${{\text{X}}}_{{{\text{A}}}_{{\text{ij}}}}^{\mathrm{^{\prime}}}$= new suspected location	rand1 & rand2 are random numbers in the range [0, 1]
${{\text{p}}}_{{{\text{A}}}_{{\text{i}}}}$= possibility that the suspect is at location ${{\text{X}}}_{{{\text{A}}}_{{\text{i}}}}$	$\mathrm{\alpha }$ = effectiveness coefficient i.e. [− 1, 1]
${{\text{p}}}_{{\text{min}}}=$ highest possibility position corresponding to the best objective value	d, k, h, and i are four suspected locations, {d,k, h, i} ϵ {1, 2, …, NP},d, k and h are chosen randomly ; NP is the number of suspected locations
${{\text{p}}}_{{\text{max}}}=$ lowest possibility value corresponding to the worst objective value	j = 1, 2, …, D; D is the number of dimensions
${{\text{X}}}_{{\text{min}}}=$ highest possibility position corresponding to the best solution	${{\text{a}}}_{1} \& {{\text{a}}}_{2}$ are number of individuals that affect the movement of ${{\text{X}}}_{{{\text{A}}}_{{\text{ij}}}}$ assumed to be 2 & 3

### Conventional arithmetic optimization algorithm (CAOA)

**Figure 4:**
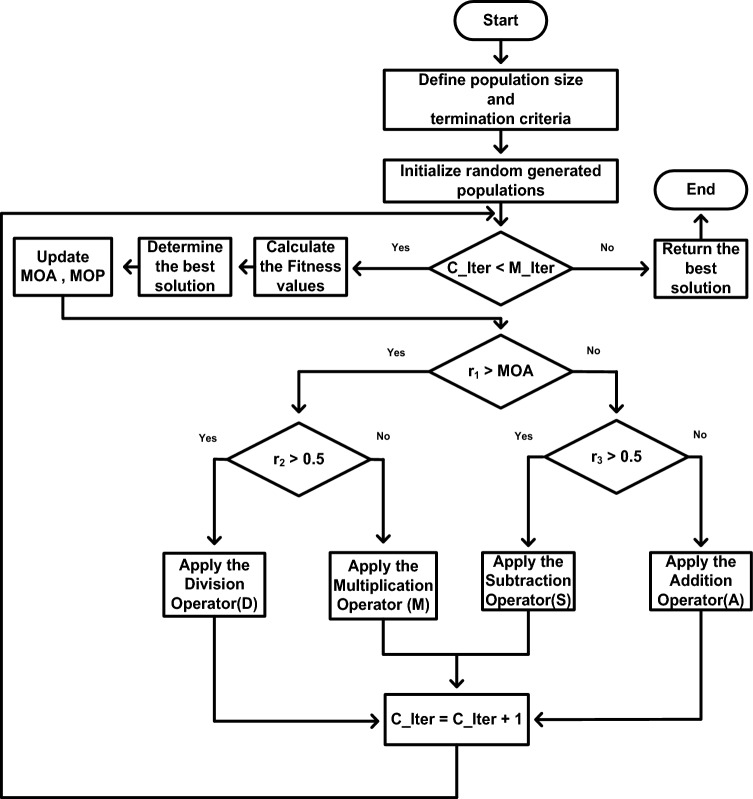
Flowchart of CAOA.

Abualigah et al.^33^ proposed the CAOA as a new meta-heuristic methodology in 2021. To achieve a globally optimized solution, the position updating equations embody four conventional arithmetic operators: addition (A), subtraction (S), multiplication (M), and division (D). For the exploratory search, multiplication (M) and division (D) are utilized, providing a large step in the search space in accordance with the various impacts of these four arithmetic operators. In order to carry out the exploitation search, which can yield small step sizes in the search space, addition (A) and subtraction (S) are performed. Figure 4 depicts the CAOA optimisation technique.

i. Phase of initialization

To choose between exploitation and exploration, the Math Optimizer Accelerated (MOA) function is calculated. The following is the function:

26 $${\text{MOA}}={{\text{min}}}_{{\text{a}}}+{\text{iter}}\times \left(\frac{{{\text{max}}}_{{\text{a}}}-{{\text{min}}}_{{\text{a}}}}{{{\text{iter}}}_{{\text{max}}}}\right)$$

where ‘iter’ and ‘iter_max_’ are the number of iterations and the maximum number of iterations, respectively. ‘min_a_’ and ‘max_a_’ are the accelerated function's minimum and maximum values, which are 0.2 and 0.9, respectively.

ii. Exploration stage

To randomly explore the area, the operators Multiplication ("X") and Division (" ÷ ") are employed. By generating the three random numbers r_1_, r_2_, and r_3_, the solution is updated.

if r_1_ < MOA.

if r_2_ > 0.5

27 $$ {\text{x}}_{{{\text{new}}}} = {\text{g}}_{{\text{best }}} \div \left( {{\text{MOP}} + {\upvarepsilon }} \right) \times \left( {\left( {{\text{U}}_{{\text{l}}} - {\text{L}}_{{\text{l}}} } \right) \times {\upmu } + {\text{L}}_{{\text{l}}} } \right) $$

else

28 $$ {\text{x}}_{{{\text{new}}}} = {\text{g}}_{{{\text{best}}}} \times {\text{MOP}} \times \left( {\left( {{\text{U}}_{{\text{l}}} - {\text{L}}_{{\text{l}}} } \right) \times {\upmu } + {\text{L}}_{{\text{l}}} } \right) $$

end.

Equation (29) represents the Math Optimizer Probability (MOP) coefficient.

29 $${\text{MOP}}=1-\frac{{\left({\text{iter}}\right)}^{\frac{1}{\mathrm{\alpha }}}}{{\left({{\text{iter}}}_{{\text{max}}}\right)}^{\frac{1}{\mathrm{\alpha }}}}$$

where g_best_ denotes the global optimum solution, $\upvarepsilon$ is a small integer value that prevents division from occurring when the denominator is zero, ${{\text{U}}}_{{\text{l}}}$ and ${{\text{L}}}_{{\text{l}}}$ represents the upper and lower limits of every dimension, $\upmu$ is equal to 0.5, and α is a sensitive parameter taken as 5.

iii. Exploitation Stage

The exploitation is carried out by the mathematical operators such as subtraction ("-") and addition (“ + ”). The steps associated with the exploitation phase are as follows:

if r_3_ < 0.5

30 $${{\text{x}}}_{{\text{new}}}={{\text{g}}}_{{\text{best}}}-{\text{MOP}}\times \left(\left({{\text{U}}}_{{\text{l}}}-{{\text{L}}}_{{\text{l}}}\right)\times\upmu +{{\text{L}}}_{{\text{l}}}\right)$$

else

31 $${{\text{x}}}_{{\text{new}}}={{\text{g}}}_{{\text{best}}}+{\text{MOP}}\times \left(\left({{\text{U}}}_{{\text{l}}}-{{\text{L}}}_{{\text{l}}}\right)\times\upmu +{{\text{L}}}_{{\text{l}}}\right)$$

end.

### Improved arithmetic optimization algorithm (IAOA)

i. Inspiration for improving the CAOA
  According to the optimum global solution, the population is updated in CAOA. As soon as the population reaches the optimum region, it will begin to stagnate. Premature coverage occasionally occurs. Additionally, the individual data of the population is not fully utilized by this method. Therefore, this paper presents an IAOA to make the most of the exploitation of individual data and address the boundaries acknowledged in CAOA.
ii. Mechanism for the proposed algorithm
  A hybrid optimization technique is presented, coalescing the strong point of PSO and CAOA.

**Figure 5:**
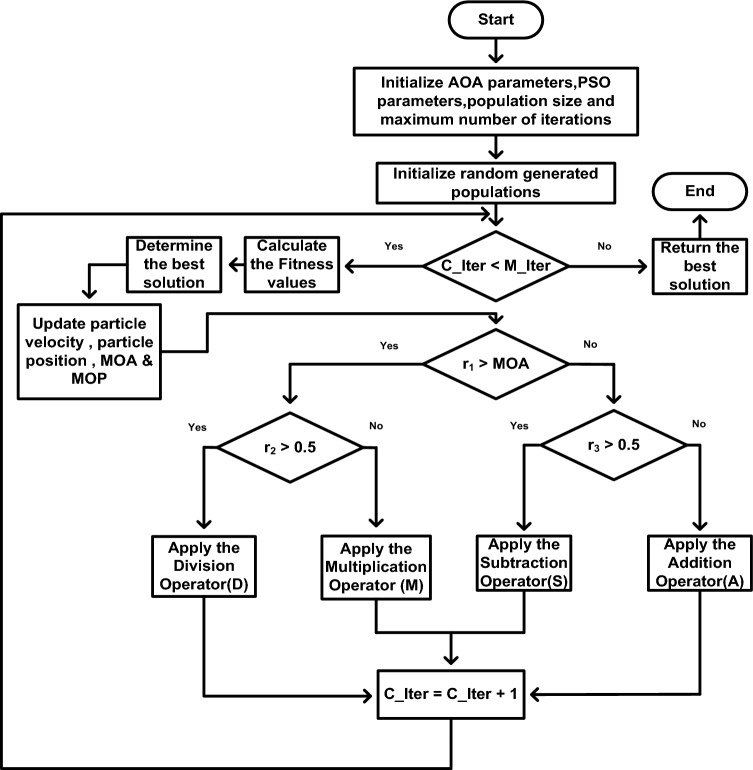
Flowchart of IAOA.

The flowchart of IAOA procedure is shown in Fig. 5. The parameters ‘α’ and ‘μ’ are taken as 5 and 0.5, respectively. The procedural code sequence for IAOA is defined as follows:

i. Determine the initial population of the design constants and design variables 'α' and 'μ' employed in the CAOA methodology.
ii. By assessing the objective function, find the solution that performs the best (g_best_).
iii. Update the solution using Eqs. (26–31) of the CAOA technique.
iv. The values of ‘α’ and ‘μ’ are updated using the Eqs. (16–17) of the PSO technique.
v. Until the terminating condition is reached, the previous two steps must be continued.

**Figure 6:**
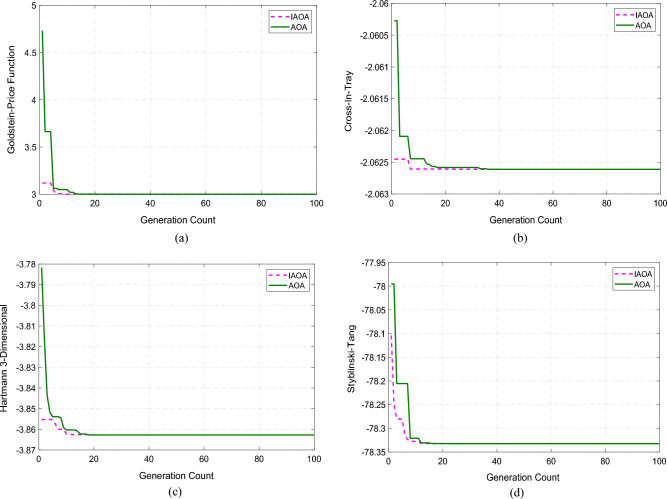
Convergence plots of benchmark functions. (**a**) Convergence characteristics of Goldstein-Price function. (**b**) Convergence characteristics of Cross-In-Tray function. (**c**) Convergence characteristics of Hartmann 3-Dimensional function. (**d**) Convergence characteristics of Styblinski-Tang function.

In order to prove the supremacy of IAOA over CAOA, this article analyses four benchmark functions, including Goldstein-Price, Cross-In-Tray, Hartmann 3-Dimensional, and Styblinski-Tang^56^. The presentation, size, and scope of each distinct function are displayed in Table 2. Both IAOA and CAOA were implemented and performed using MATLAB with a population and iteration limit of 100. Table 3 presents performance metrics that demonstrate the higher performance of IAOA, including mean, maximum, minimum, and standard deviation. The IAOA method takes less iteration to attain the global optimal value, however, the IAOA algorithm's computation time is a little bit longer due to the additional updating step. Figure 6 displays the convergence plots for various benchmark functions.

**Table 2:** Description of the benchmark functions employed in the study.

Function	Function’s Expression	Dimension	Range
Goldstein-Price (F_1_)	$$\begin{aligned} &{\text{f}}\left( {\text{x}} \right) = \left[ {1 + \left( {{\text{x}}_{1} + {\text{x}}_{2} + 1} \right)^{2} } \right. \hfill \\ &\left. {\left( {19 - 14{\text{x}}_{1} + 3{\text{x}}_{1}^{2} - 14{\text{x}}_{2} + 6{\text{x}}_{1} {\text{x}}_{2} + 3{\text{x}}_{2}^{2} } \right)} \right] \hfill \\& *\left[ {30 + \left( {2{\text{x}}_{1} - 3{\text{x}}_{2} } \right)^{2} } \right. \hfill \\ &\left. {\left( {18 - 32{\text{x}}_{1} + 12{\text{x}}_{1}^{2} + 48{\text{x}}_{2} - 36{\text{x}}_{1} {\text{x}}_{2} + 27{\text{x}}_{2}^{2} } \right)} \right] \hfill \\ \end{aligned}$$	2	[− 2, 2]
Cross-In-Tray(F_2_)	${\text{f}}\left({\text{x}}\right)=-0.0001{\left(\left|{\text{sin}}\left({{\text{x}}}_{1}\right){\text{sin}}\left({{\text{x}}}_{2}\right){\text{exp}}\left(\left|100-\frac{\sqrt{{{\text{x}}}_{1}^{2}+{{\text{x}}}_{2}^{2}}}{\uppi }\right|\right)\right|+1\right)}^{0.1}$	2	[− 10, 10]
Hartmann 3-Dimensional (F_3_)	${\text{f}}\left({\text{x}}\right)=-\sum_{{\text{i}}=1}^{4}{{\text{a}}}_{{\text{i}}}{\text{exp}}\left(-\sum_{{\text{j}}=1}^{3}{{\text{A}}}_{{\text{ij}}}{\left({{\text{x}}}_{{\text{j}}}-{{\text{P}}}_{{\text{ij}}}\right)}^{2}\right)$	3	[0, 1]
Styblinski-Tang (F_4_)	${\text{f}}\left({\text{x}}\right)=\frac{1}{2}\sum_{{\text{i}}=1}^{{\text{d}}}\left({{\text{x}}}_{{\text{i}}}^{4}-16{{\text{x}}}_{{\text{i}}}^{2}+5{{\text{x}}}_{{\text{i}}}\right)$	d	[− 5, 5]

**Table 3:** Analysis of the CAOA, SOS, SPBO, GWO, SHADE, FA and IAOA algorithms performance.

Algorithm	Function	Optimum Value	Minimum	Maximum	Mean	Standard Deviation	Computational Time (s)
IAOA	F1	3	3	3	3	1.7021 × 10–15	0.0858
CAOA			3	3	3	7.5106 × 10–14	0.0510
SOS			3	3	3	8.235 × 10^–12^	0.1275
SPBO			3	3	3	9.7352 × 10^–12^	0.0523
GWO			3	3	3	8.5723 × 10^–11^	0.0621
SHADE			3	3	3	6.7325 × 10^–11^	0.0763
FA			3	3	3	5.5592 × 10^–10^	0.0782
IAOA	F2	− 2.0626	− 2.0626	− 2.0626	− 2.0626	1.0878 × 10–15	0.1064
CAOA			− 2.0626	− 2.0626	− 2.0626	1.7904 × 10–9	0.0615
SOS			− 2.0626	− 2.0626	− 2.0626	2.2658 × 10^–9^	0.1257
SPBO			− 2.0626	− 2.0626	− 2.0626	5.5537 × 10^–8^	0.0879
GWO			− 2.0626	− 2.0626	− 2.0626	6.6235 × 10^–8^	0.0767
SHADE			− 2.0626	− 2.0626	− 2.0626	8.2301 × 10^–7^	0.0872
FA			− 2.0626	− 2.0626	− 2.0626	7.2156 × 10^–6^	0.0975
IAOA	F3	− 3.8628	− 3.8628	− 3.8628	− 3.8628	3.1618 × 10–15	0.1057
CAOA			− 3.8628	− 3.8628	− 3.8628	1.7828 × 10–12	0.0586
SOS			− 3.8628	− 3.8628	− 3.8628	3.2357 × 10^–11^	0.1527
SPBO			− 3.8628	− 3.8628	− 3.8628	5.5867 × 10^–11^	0.0623
GWO			− 3.8628	− 3.8628	− 3.8628	7.2314 × 10^–10^	0.0789
SHADE			− 3.8628	− 3.8628	− 3.8628	9.0539 × 10^–10^	0.0843
FA			− 3.8628	− 3.8628	− 3.8628	5.2788 × 10^–09^	0.0872
IAOA	F4	− 78.3323	− 78.3323	− 78.3323	− 78.3323	1.4454 × 10–14	0.0749
CAOA			− 78.3323	− 78.3323	− 78.3323	1.5365 × 10–13	0.0384
SOS			− 78.3323	− 78.3323	− 78.3323	5.5605 × 10^–13^	0.1273
SPBO			− 78.3323	− 78.3323	− 78.3323	8.2309 × 10^–13^	0.0589
GWO			− 78.3323	− 78.3323	− 78.3323	6.3058 × 10^–12^	0.0695
SHADE			− 78.3323	− 78.3323	− 78.3323	8.8901 × 10^–12^	0.0789
FA			− 78.3323	− 78.3323	− 78.3323	6.6201 × 10^–11^	0.0802

## Design of controllers

Any control system must have controllers to minimize the difference between measured and actual values. To regulate active and reactive power, the controllers ensure that the actual currents align with the specified references. Numerous controllers are employed for controlling electrical power, such as PI, SMC and ST-SMC.

### Proportional integral (PI) controller

A PI controller blends both integral and proportional control actions^57^. By comparing the system output to the set point, a feedback control loop can determine the error signal^58^. The PI controller's mathematical expression is provided in (32).

32 $${\text{Output}}\left({\text{t}}\right)={\text{e}}\left({\text{t}}\right)+{{\text{K}}}_{{\text{i}}}{\int }_{0}^{{\text{t}}}{\text{e}}\left({\text{t}}\right){\text{d}}({\text{t}})$$

where e(t) is an error, K_p_ is the proportional gain, and K_i_ is the integral gain.

Using the above equation's Laplace transform,

33 $${\text{Output}}\left({\text{t}}\right)={\text{E}}({\text{s}})\left[{{\text{K}}}_{{\text{p}}}+\frac{{{\text{K}}}_{{\text{i}}}}{{\text{s}}}\right]$$

### Sliding mode controller (SMC)

The SMC is a variable structure based control technique in which the system states are driven towards the sliding surface with a switching logic S(x) alternating between two limit values^59^. The process essentially consists of two steps: sliding in the direction of the surface and subsequent convergence towards the surface^60^.

The sliding mode controller design is carried out in three stages:

i. Decision of the sliding surface.
ii. Establishment of the existence condition of sliding mode.
iii. Design of the control law.

i. Decision of the sliding surface:

A non-linear system is taken into account as shown in Eq. (34):

34 $$ \left\{ {\begin{array}{*{20}l} {{\text{x}}^{{\text{n}}} = f\left( {{\text{x}},{\text{t}}} \right) + g\left( {{\text{x}},{\text{t}}} \right) u \left( {{\text{x}},{\text{t}}} \right)} \hfill \\ {x \in {\text{R}}^{{\text{n}}} , u \in R} \hfill \\ \end{array} } \right. $$

As proposed by Slotine and Li^61^, we take the following general equation for sliding surface:

35 $${\text{S}}\left({\text{x}}\right)={\left(\frac{{\text{d}}}{{\text{dt}}}+\uplambda \right)}^{\left({\text{n}}-1\right)}{\text{e}}({\text{x}})$$

36 $${\text{e}}\left({\text{x}}\right)={\text{x}}-{{\text{x}}}^{*}$$

where ‘n’ stands for the sliding mode controller's order, ‘x’ stands for the state that needs to be controlled, ‘λ’ stands for a positive constant that determines the system's bandwidth and ‘x*’ for the desired state, 'f 'and 'g' are uncertain supposed bounded and continuous.

ii. Establishment of the existence condition of sliding mode:

The Lyapunov function is taken into consideration for determining the attractiveness condition:

37 $${\text{V}}\left({\text{S}}\right)=\frac{1}{2}{{\text{S}}}^{2}$$

The sliding variable S(x, t) tends to be zero when the derivative of V(S) is negative, which defines the attractiveness condition.

38 $${\text{S}}.{\dot{\text{S}}} < 0$$

A more restrictive condition known to be η-attractively is used instead of the condition in Eq. (38), which ensures asymptotic convergence in the direction of the sliding surface for convergence in finite time.

39 $$ {\dot{\text{S}}}.{\text{S}} \le - {\upeta }\left| {\text{S}} \right|\;\;{\text{Where }}\;\;\eta \, > \,0 $$

iii. Design of the control law:

The controlled variable S = 0 is kept on the sliding surface when the equivalent command is a continuous function. S = 0 and ${\dot{\text{S}}}=0$ are the conditions of invariance^62^. On the other hand, this order does not compel the system’s trajectories to converge in the direction of the sliding surface. The sum of the discontinuous component (u_d_) and the equivalent command (u_eq_) provides the command u.

40 $${\text{u}}={{\text{u}}}_{{\text{eq}}}+{{\text{u}}}_{{\text{d}}}$$

41 $${{\text{u}}}_{{\text{d}}}=-{\text{K}}*{\text{Sign}}({\text{S}})$$

In this case, the constant "K" is positive. The chattering effect is produced by Eq. (41). Oscillations caused by chattering in electro-mechanical systems may result in warmth. The discontinuous component in Eqs. (42) and (43) can be expressed as functions to reduce chattering.

42 $${{\text{u}}}_{{\text{d}}}=-{\text{K}}*{\text{tanh}}\left({\text{S}}\right)$$

43 $$ {\text{u}}_{{\text{d}}} = - {\text{K*Sat}}\left( {\frac{{\text{S}}}{\upphi }} \right) $$

where,

44 $$ {\text{Sat}}\left( {\frac{{\text{S}}}{\upphi }} \right) = \left\{ {\begin{array}{*{20}l} 1 \hfill & {{\text{for S}} >\upphi } \hfill \\ {\frac{{\text{S}}}{\upphi }} \hfill & {{\text{for}} -\upphi < S <\upphi } \hfill \\ { - 1} \hfill & {{\text{for S}} < -\upphi } \hfill \\ \end{array} } \right. $$

where ‘ϕ’ stands for the width of the boundary layer. The chattering is reduced when ‘sign(S)’ is changed to ${\text{Sat}}\left( {\frac{{\text{S}}}{\upphi }} \right)$.

### High-order sliding mode

The alternative approach to the chattering problems from the conventional sliding mode control technique is resolved by adopting the theory of higher-order sliding modes^63^. This method reduces chattering by removing the discontinuous term directly from the synthesised command and putting it instead into one of its higher derivatives^64,65^. Beyond improving asymptotic accuracy, high-order sliding modes have been developed to address chattering difficulties while retaining the convergence characteristics and durability typical of ordinary sliding mode controllers. These methods frequently rely on the concept of homogeneity and a set of coefficients or weights.

### Super-twisting sliding mode controller

i. Twisting algorithm

Aligned with the quadrant in which the system's state is positioned, not only is the sign of the control switched, but the amplitude also alternates between two values. In the phase plane, the trajectory of the system spirals towards the origin while navigating around it^66^. Equation (45) presents the system of relative degree 2. The robustness and stability of the control system are largely dependent on Eqs. (45) and (46). In order to minimize the effects of uncertainties and disturbances, the algorithm is designed to produce control signals that move the system towards the target trajectory. In particular, following the reference trajectory and rejecting disturbances are made easier when the twisting algorithm is applied throughout the entire algorithm. The twisting algorithm does this by continuously modifying the control input in response to the difference between the system's actual state and the intended trajectory. Fast convergence and robustness depend on the information regarding the control signal's rate of change.

**Figure 7:**
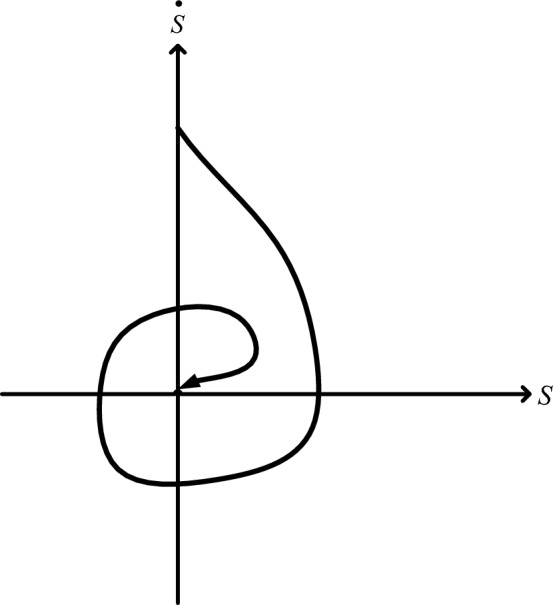
Convergence of the twisting algorithm in the plane(S,$\dot{{\text{S}}}$).

Moreover, chattering is avoided and smooth control action is ensured by the twisting method, which also guarantees that the control signal stays restricted. To do this, a non-linear term that smoothes transitions between various control actions by acting as a smoothing factor is added to the control rule. Overall, by ensuring stability, fast convergence, and robustness towards the intended trajectory, the twisting algorithm in the super-twisting sliding mode controller greatly enhances the overall performance of the control system. Figure 7 presents the convergence of the twisting algorithm in the plane(S, $\dot{{\text{S}}}$).

45 $${\text{u}}=-{{\text{r}}}_{1}{\text{sign}}\left({\text{S}}\right)-{{\text{r}}}_{2}{\text{sign}}\left({\text{S}}\right), {{\text{r}}}_{2}>{{\text{r}}}_{1}>0$$

46 $$ \left\{ {\begin{array}{*{20}l} {\left( {{\text{r}}_{1} + {\text{r}}_{2} } \right){\text{K}}_{{\text{m}}} - {\text{C}}_{0} > \left( {{\text{r}}_{1} - {\text{r}}_{2} } \right){\text{K}}_{{\text{M}}} + {\text{C}}_{0} } \hfill \\ {\left( {{\text{r}}_{1} - {\text{r}}_{2} } \right){\text{K}}_{{\text{m}}} > {\text{C}}_{0} } \hfill \\ \end{array} } \right. $$

The control law is homogeneous as it does not depend on the sign of S or $\dot{{\text{S}}}$ when when multiplied by K > 0.

ii. Algorithm of super-twisting

In the realm of second-order sliding mode control, the super-twisting algorithm stands out as an exception. Originally designed for systems possessing a relative degree of 1 concerning a sliding surface, this algorithm offers unique capabilities^67^. Super twisting algorithm is advantageous because it does not utilize this information. It is composed of two parts: a continuous part u1 and a discontinuous part^67^.

47 $${\text{u}}\left({\text{t}}\right)={{\text{u}}}_{1}\left({\text{t}}\right)+{{\text{u}}}_{2}\left({\text{t}}\right)$$

48 $$ {\dot{\text{u}}}_{1} = \left\{ {\begin{array}{*{20}l} { - {\text{u}}\;\;{\text{if}} \left| {\text{u}} \right| > {\text{U}}_{{\text{M}}} } \hfill \\ { - \alpha {\text{sign}}\left( {\text{S}} \right){ }\;\;{\text{if}}\; {\text{not}}} \hfill \\ \end{array} } \right. $$

49 $$ {\text{u}}_{2} = \left\{ {\begin{array}{*{20}l} { -\uplambda \left| {{\text{S}}_{0} } \right|^{{\uprho }} {\text{sign}}\left( {\text{S}} \right)\;\;{\text{ if}} \left| {\text{u}} \right| > {\text{S}}_{0} } \hfill \\ { -\uplambda \left| {\text{S}} \right|^{{\uprho }} {\text{sign}}\left( {\text{S}} \right) \;\;{\text{if}} {\text{not}}} \hfill \\ \end{array} } \right. $$

With $\uprho$, λ, α checking for the following inequalities:

50 $$ \left\{ {\begin{array}{*{20}l} {\upalpha  > \frac{{{\text{C}}_{0} }}{{{\text{K}}_{{\text{m}}} }}, \;\;0 < \rho < 0.5} \hfill \\ {{\uplambda }^{2} = \frac{{4{\text{C}}_{0} {\text{K}}_{{\text{M}}} \left( {{\upalpha } + {\text{C}}_{0} } \right)}}{{{\text{K}}_{{\text{m}}}^{2} {\text{K}}_{{\text{m}}} \left( {{\upalpha } - {\text{C}}_{0} } \right)}}} \hfill \\ \end{array} } \right. $$

where K_M_, K_m_, and C_0_ are positive coefficients. We can make the process simpler if ${{\text{S}}}_{0}=\infty$

51 $$ \left\{ {\begin{array}{*{20}l} {{\text{u}} = -\uplambda \left| {\text{S}} \right|^{{\uprho }} {\text{sign}}\left( {\text{S}} \right) + {\text{u}}_{1} } \hfill \\ {{\dot{\text{u}}}_{1} = - \alpha {\text{sign}}\left( {\text{S}} \right)} \hfill \\ \end{array} } \right. $$

where the sliding mode parameter, super twisting variable and control input are represented by λ, ${\dot{{\text{u}}}}_{1}$ and u, respectively. In this article, ${\text{Sat}}\left( {\frac{{\text{S}}}{\upphi }} \right)$ in (44) is used in instead of the switching function ‘sign(S)’ in (51).

To regulate both active and reactive power, Fig. 2 incorporates two control loops. The initial loop oversees the active power, while the second loop manages the reactive power^68^.

52 $$ \begin{aligned} {\text{S}}\left( {\text{P}} \right) & = {\upbeta }_{{\text{P}}} {\text{e}}\left( {\text{P}} \right) \\ {\text{S}}\left( {\text{Q}} \right) & = {\upbeta }_{{\text{Q}}} {\text{e}}\left( {\text{Q}} \right) \\ \end{aligned} $$

where,

53 $$ \begin{aligned} {\text{e}}\left( {\text{P}} \right) & = {\text{P}}^{*} - {\text{P}} \\ {\text{e}}\left( {\text{Q}} \right) & = {\text{Q}}^{*} - {\text{Q}} \\ \end{aligned} $$

Equation (46) specifies the amplitudes of the reference in-phase and quadrature currents.

54 $$ \begin{aligned} {\text{I}}_{{{\text{mP}}}}^{*} & = - {\uplambda }_{{\text{P}}} \left| {{\text{S}}\left( {\text{P}} \right)} \right|^{{{\uprho }_{{\text{P}}} }} {\text{Sat}}\left( {\frac{{{\text{S}}\left( {\text{P}} \right)}}{{\Phi_{{\text{P}}} }}} \right) + \smallint - {\upalpha }_{{\text{P}}} {\text{Sat}}\left( {\frac{{{\text{S}}\left( {\text{P}} \right)}}{{\Phi_{{\text{P}}} }}} \right) \\ {\text{I}}_{{{\text{mQ}}}}^{*} & = - {\uplambda }_{{\text{Q}}} \left| {{\text{S}}\left( {\text{Q}} \right)} \right|^{{{\uprho }_{{\text{Q}}} }} {\text{Sat}}\left( {\frac{{{\text{S}}\left( {\text{Q}} \right)}}{{\Phi_{{\text{Q}}} }}} \right) + \smallint - {\upalpha }_{{\text{Q}}} {\text{Sat}}\left( {\frac{{{\text{S}}\left( {\text{Q}} \right)}}{{\Phi_{{\text{Q}}} }}} \right) \\ \end{aligned} $$

## Results and discussion

**Figure 8:**
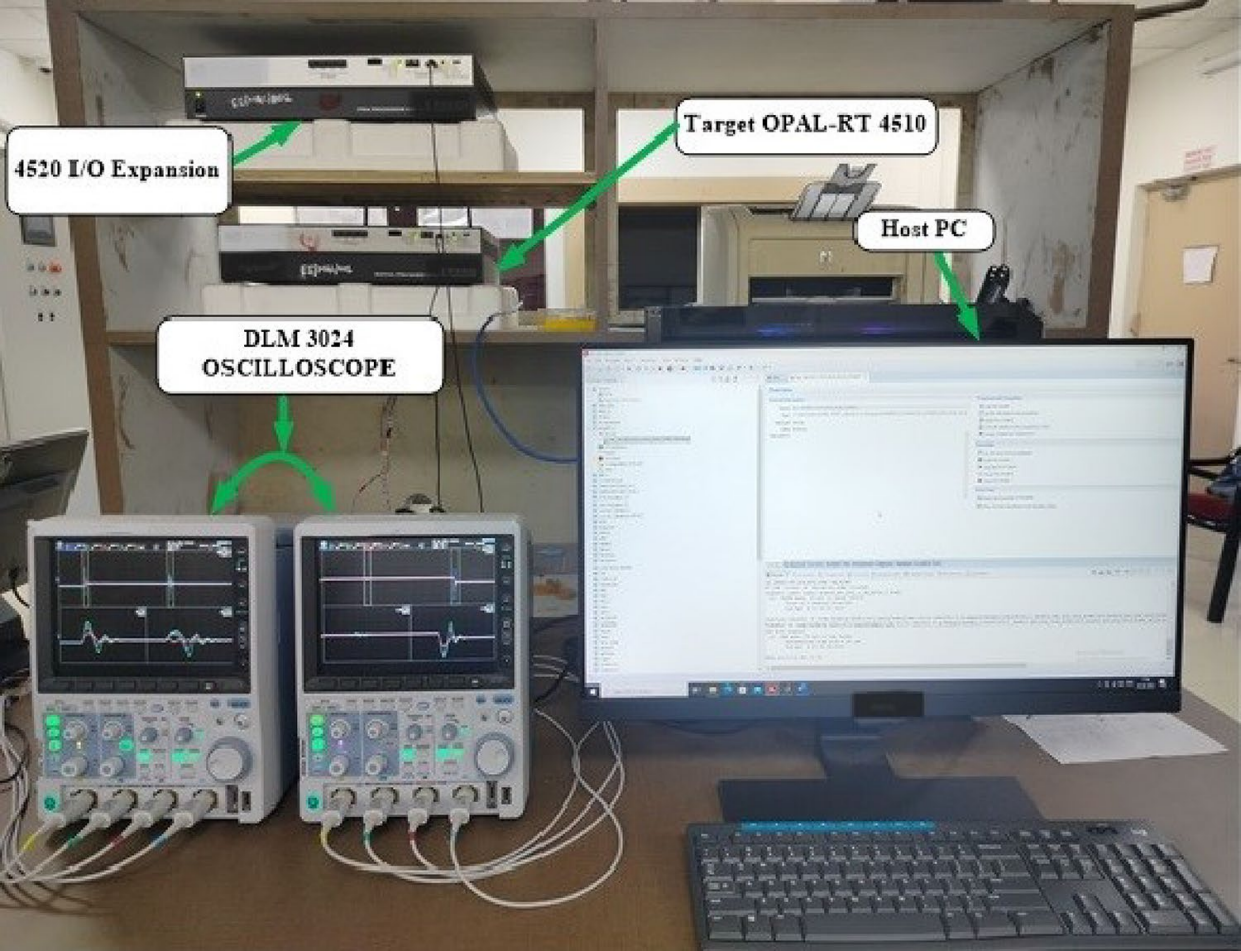
Real-time experimental setup using OPAL-RT.

The efficacy of a grid-connected PV system experiences enhancement through the utilization of an optimally tuned ST-SMC proposed in this article. This section entails a comparison and presentation of system performances for each of the three different algorithms incorporating ST-SMC. Additionally, the bidirectional converter enables a two-way flow of power, allowing the 40 kW PV system to supply electricity to the grid while simultaneously meeting the needs of a local load. The grid can get active as well as reactive power support from the bidirectional converter, which is managed by a P–Q control framework. Both active and reactive power can be independently controlled using the PQ control framework. The active power reference changes stepwise from 12 to 20 kW while maintaining a unity power factor. Similarly, the reactive power varies stepwise from '0' kVAR to 14 kVAR. The reference's negative sign indicates that the power is sent to the grid. The load is regarded to be constant throughout the whole process. The ST-SMC controller is tuned using three different algorithms such as PSO, CAOA and IAOA techniques, for the performance comparison. Table 4 lists the gains of various algorithms based ST-SMC technique. Figure 8 depicts the real-time experimental set-up implemented in OPAL-RT 4510. The dynamic performance of the optimally tuned ST-SMC technique is presented in Table 5. Table 6 summarises the real-time parameters used in the experiments, providing insights into the important requirements governing system dynamics and performance during real-time simulations.

**Table 4:** Gains of various algorithms based on ST-SMC technique.

Parameters governing active power control Type of optimization-based controller						Parameters governing reactive power controlType of optimization-based controller					
PSO-STSMC		CAOA-STSMC		IAOA-STSMC		PSO-STSMC		CAOA-STSMC		IAOA-STSMC	
K_p_	2	K_p_	2.53	K_p_	3	K_p_	2	K_p_	2	K_p_	3
K_s_	50	K_s_	60	K_s_	56	K_s_	50	K_s_	50	K_s_	56
K_T_	0.4	K_T_	0.4	K_T_	0.5	K_T_	0.6961	K_T_	0.6389	K_T_	0.5
alpha	0.6723	alpha	0.6235	alpha	0.5	alpha	0.5904	alpha	0.6172	alpha	0.5
K	0.5904	K	1	K	1	K	0.7	K	0.7	K	1
phi	19.17	phi	19.49	phi	16	phi	20	phi	20	phi	16

**Table 5:** Dynamic performance of optimally tuned ST-SMC technique.

Optimization based Controller	Performance under active power control		Performance under reactive power control	
	Rise time (msec)	Settling time (sec)	Rise time (msec)	Settling time (sec)
PSO-STSMC	0.52	0.01174	0.54	0.5352
CAOA-STSMC	0.34	0.01162	0.52	0.5156
IAOA-STSMC	0.29	0.01012	0.48	0.5075

**Table 6:** Real-time parameters.

Parameter	Specifications
Type of Simulation	Real-Time
Step Time	10 μs
Simulator	OP 4510 (RCP/HIL Kintex-7 FPGA Processor)
FPGA Software	Xilinx Kintex-7 FPGA, 485 T
Operating System	Windows 10
Target Software Used	RT-Lab
Memory	32 GB

**Figure 9:**
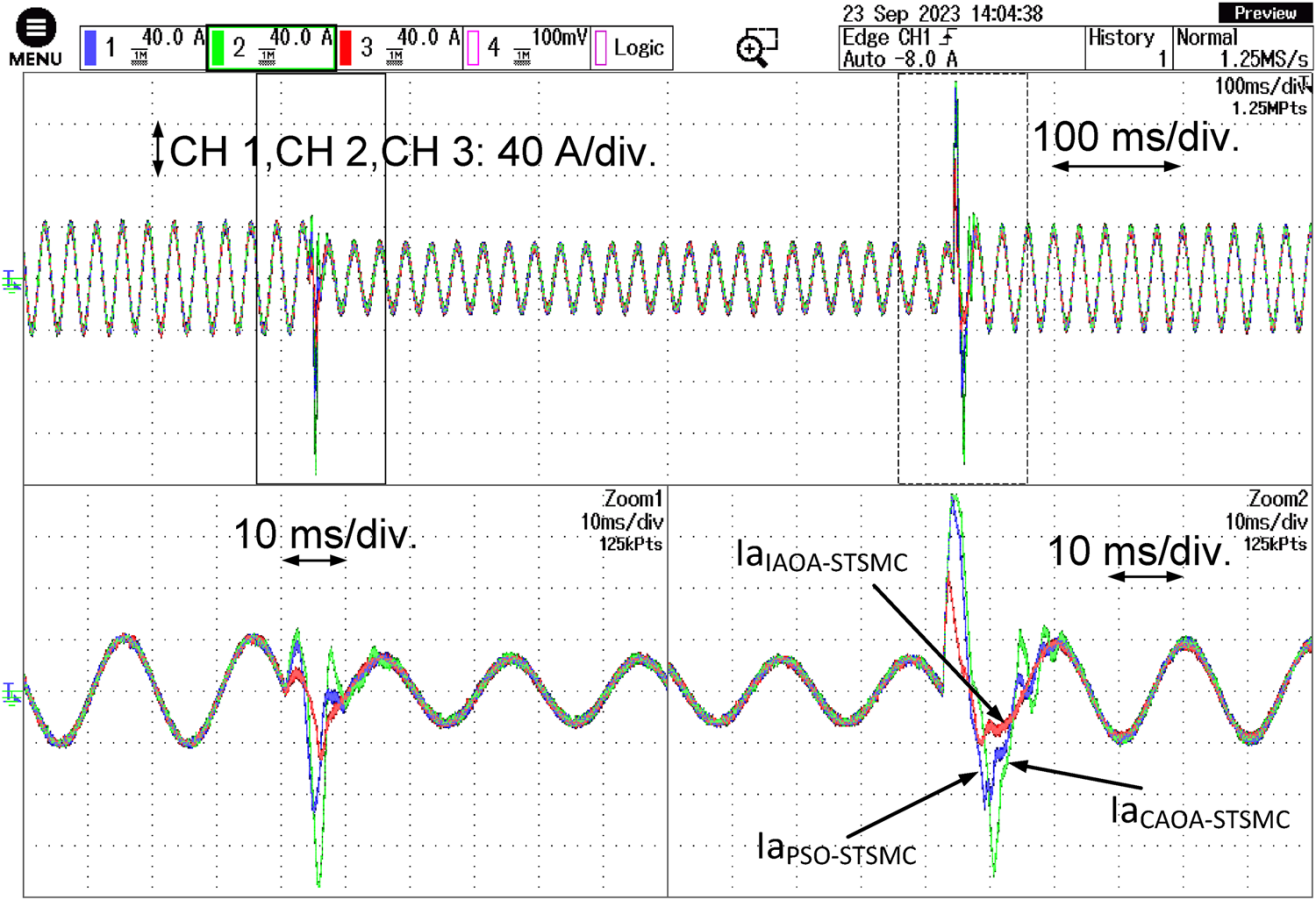
Three-phase grid current responses with the three algorithm-based ST-SMC.

Figure 9 demonstrates the system's three-phase grid current responses while using the three algorithms-based STSMC technique. A distinct decrease in current is visible in the responses with PSO-based STSMC. Similar to this, the grid current may experience a significant undershoot. The figure makes it abundantly evident that the reactive power variation has a greater impact on the PSO-based STSMC performance than the active power variation. Once more, the PSO-based STSMC exhibits a slow settling time. The CAOA-based STSMC exhibits a quicker settling time as compared to the PSO-based STSMC. As can be observed from the figure, both variations in active and reactive power have an equal impact on the CAOA-STSMC performance. The current responses with IAOA-STSMC are shown in Fig. 9. It is abundantly evident that IAOA-STSMC has the minimum settling time.

**Figure 10:**
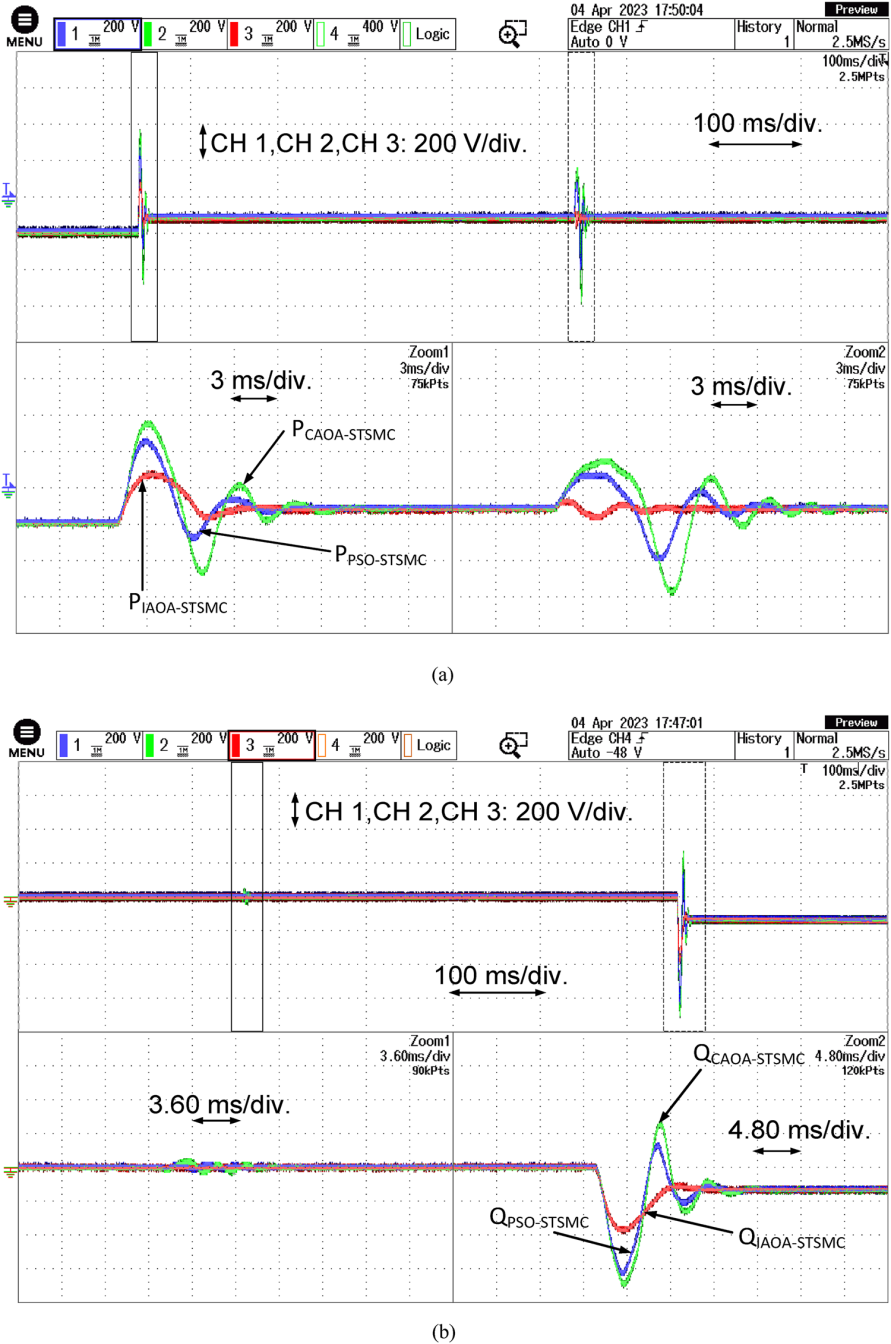
The grid side active and reactive power responses with the three algorithms based ST-SMC.

Figure 10 represents the grid-side active and reactive power responses using three algorithms based on the ST-SMC technique. Figure 10a shows that among all optimally tuned techniques, the PSO-based ST-SMC technique performs the worst with a settling time of 0.01174 s. In contrast, CAOA-based ST-SMC has a settling time of 0.01162 s, and IAOA-based ST-SMC performs best with a settling time of 0.01012 s for active power control. Figure 10b depicts the results of the three algorithms based on the ST-SMC technique for reactive power control. The IAOA-ST-SMC's supremacy is also evident in this domain. For reactive power control, the IAOA-based ST-SMC has the lowest settling time of 0.5075 s, followed by CAOA-based ST-SMC at 0.5156 s and PSO-based ST-SMC at 0.5352 s.

**Figure 11:**
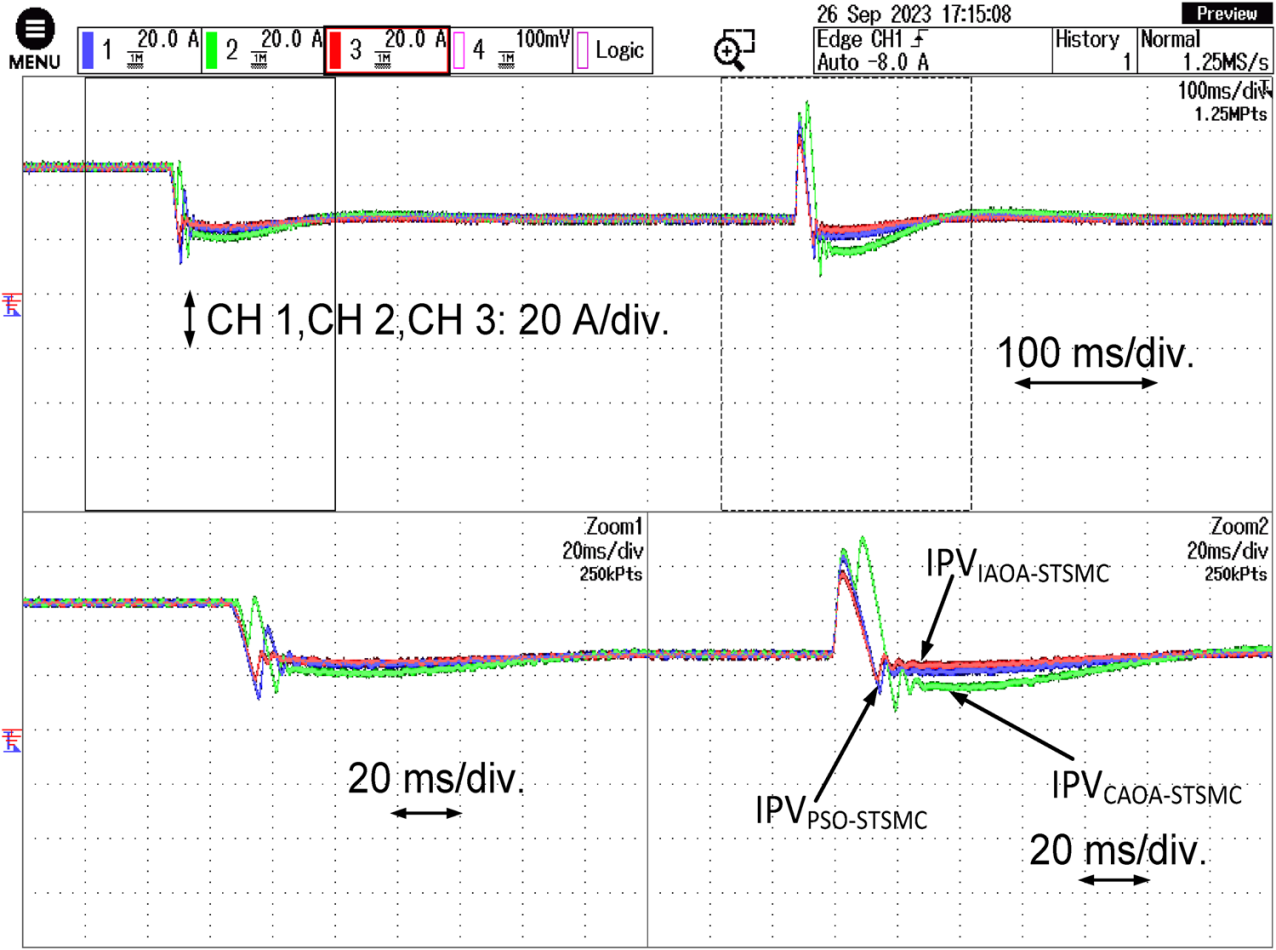
PV side current response with all the three algorithms based ST-SMC.

The PV side current response for all three algorithm-based STSMC is shown in Fig. 11. The DC side of the PSO-based PI controller exhibits the most significant effects of variations in active and reactive power references. The overshoots and undershoots for all algorithms during active power change are remarkably identical. However, the maximum settling time exists in the case of the PSO-based PI technique. The IAOA-based ST-SMC exhibits its most outstanding performance in this particular scenario as well.

## Conclusion and future research directions

The active and reactive power control for GCPV systems have been addressed in this article. Three optimization techniques constitute the basis for the ST-SMC, which controls the GCPV system's reactive and active power. The performances of different algorithms-based ST-SMC approaches are compared and investigated using disturbance for control of both active and reactive power. PI controllers are typically utilized by the GCPV system in practice. However, the results of previous studies indicate that there is a problem with a significant initial undershoot with the PI controller. Though the results show chattering issues, SMC can help overcome the constraints of PI controllers. The ST-SMC controller is distinguished by its versatility, high precision, simplicity, and fast response. The proposed IAOA-based ST-SMC appears to be superior than the PSO-based ST-SMC and the CAOA-based ST-SMC, based on a comparative analysis. The settling time for active and reactive power control are at least 0.01012 s and 0.5075 s, respectively. To support the simulation results, a performance analysis of the system is also performed in real-time using OPAL-RT.

Optimized controller design is used in the MATLAB environment to implement the three GCPV system that is proposed for controlling active and reactive power. As for the simulation and real-time validation in OPAL-RT 4510, the optimal values of the controllers remain the same; nevertheless, the hardware implementation of the proposed system presents an obstacle. Owing to the time delay circuits in the experimental system, different controller optimal values would be obtained. It is necessary to re-design the controller gains while taking the experimental circuit parameters into account because the rated values of resistors, capacitance, and inductance affect the controllers' optimal values.

We have identified some potential future improvements to MAs for optimization issues that could enhance the algorithms' performance and utility in a range of fields, such as engineering, finance, and logistics management. Hybrid optimization methods—which combine machine learning techniques and MAs—may yield more accurate and efficient solutions for difficult optimization problems involving large spaces and nonlinear objective functions. Therefore, further extensions of MAs in the areas of machine learning, specialized algorithms, and scalability may significantly increase the performance and usability of these methods in numerous domains. Additionally, this might lead to more precise, efficient, and economical solutions for difficult optimization issues.

## Data Availability

The datasets used and/or analysed during the current study available from the corresponding author on reasonable request.
